# En bloc resection of a T4B stage cancer of the hepatic flexure of the colon invading the liver, gall bladder, and pancreas/duodenum: A case report

**DOI:** 10.1002/ccr3.3455

**Published:** 2020-11-04

**Authors:** Linghou Meng, Zigao Huang, Jungang Liu, Hao Lai, Hongqun Zuo, Jiankun Liao, Yuan Lin, Weizhong Tang, Xianwei Mo

**Affiliations:** ^1^ Guangxi Clinical Research Center for Colorectal Cancer Guangxi Cancer Hospital Nanning China

**Keywords:** case report, colorectal cancer, en bloc resection, multivisceral resection

## Abstract

A T4B hepatic flexure of colon cancer that had invaded the liver, gall bladder, and pancreas/duodenum was removed through a D3 expanded right hemicolectomy + pancreaticoduodenectomy +sectional VI and VII hepatic segmentectomy.

## Graphical abstract

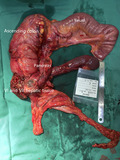

## 1 CASE REPORT

Colorectal cancer (CRC) is a highly prevalent disease worldwide, and approximately 15% of patients present with locally advanced tumors (T4 stage). En bloc resection of the tumor is of pivotal importance and is associated with a highly significant improvement in patients’ 5‐year survival. An open surgical treatment is presented for radical en bloc resection of locally advanced right colon cancer. The patient was a 54‐year‐old morbidly thin (body mass index 16.3) male who was scheduled to undergo a routine open surgery right hemicolectomy and upon exploration was found to have a large mass tumor involving segments IVb and VI of the liver, gall bladder, and duodenum. Surgical multivisceral en bloc resection was performed, and the patient went home in 14 days. The tumor was staged as T4bN2M1a, American Joint Commission on Cancer stage T4b with 27 positive nodes. En bloc resection for T4B patients has an acceptable survival rate of morbidity and mortality. In order to achieve a better oncologic outcome, multidisciplinary teamwork and multi‐modal treatment regimens can be utilized and the procedure should be undertaken by a Noticed experienced surgeon.

Colorectal cancer (CRC) is a highly prevalent disease worldwide, and approximately 15% of patients present with locally advanced tumors (T4 stage).^1^ Although there are well‐established screening techniques for colorectal cancer, it is still common for patients with colon cancer to present as locally advanced. In these cases, an en bloc multivisceral resection（MVR）of the involved organs with negative margins has been shown to reduce local recurrence and improve overall survival,^2^ we describe our technique for en bloc multivisceral resection of a locally advanced right colon cancer.

**Figure 1:**
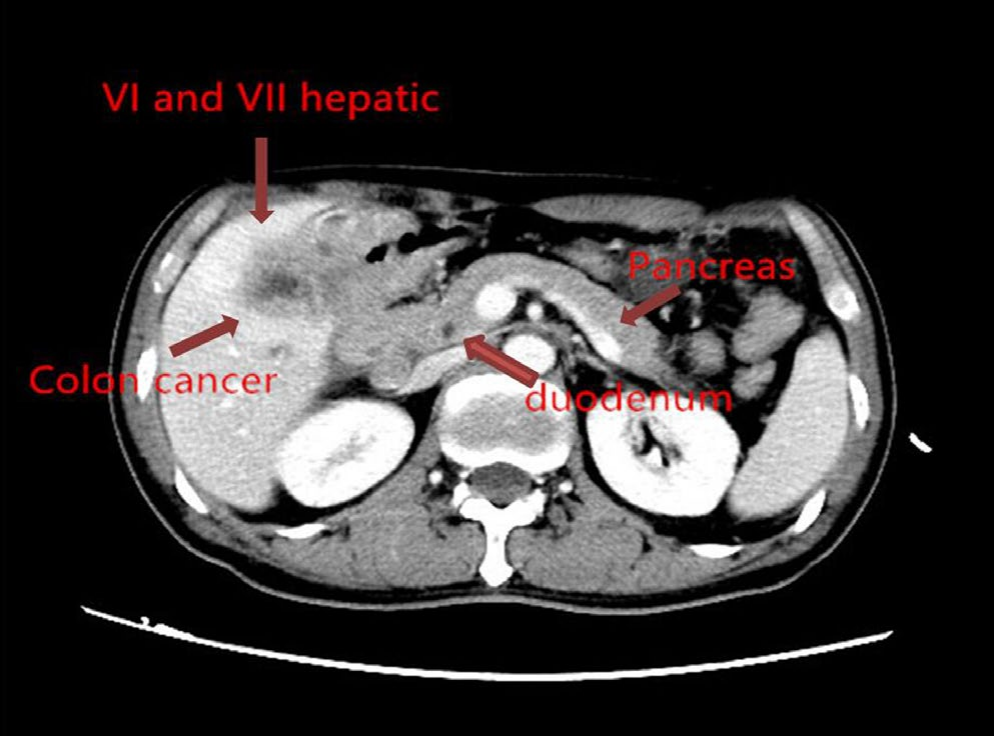
Colorectal cancer invading the liver, gall bladder, and pancreas/duodenum

**Figure 2:**
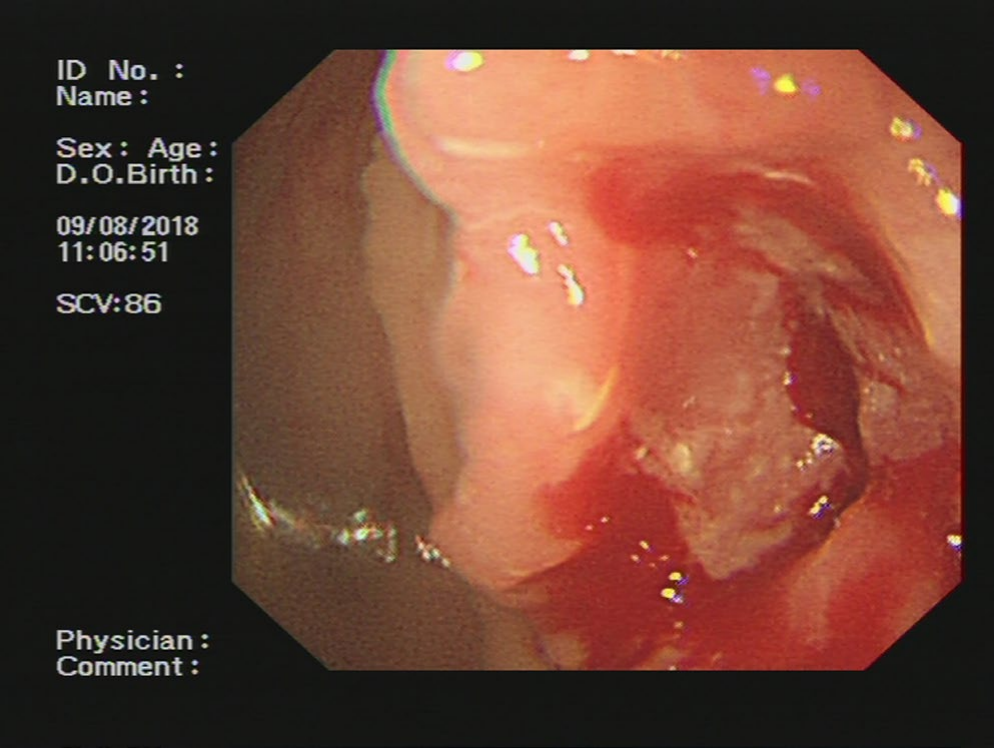
Colonoscopy identified a tumor located in the hepatic flexure of the colon

**Figure 3:**
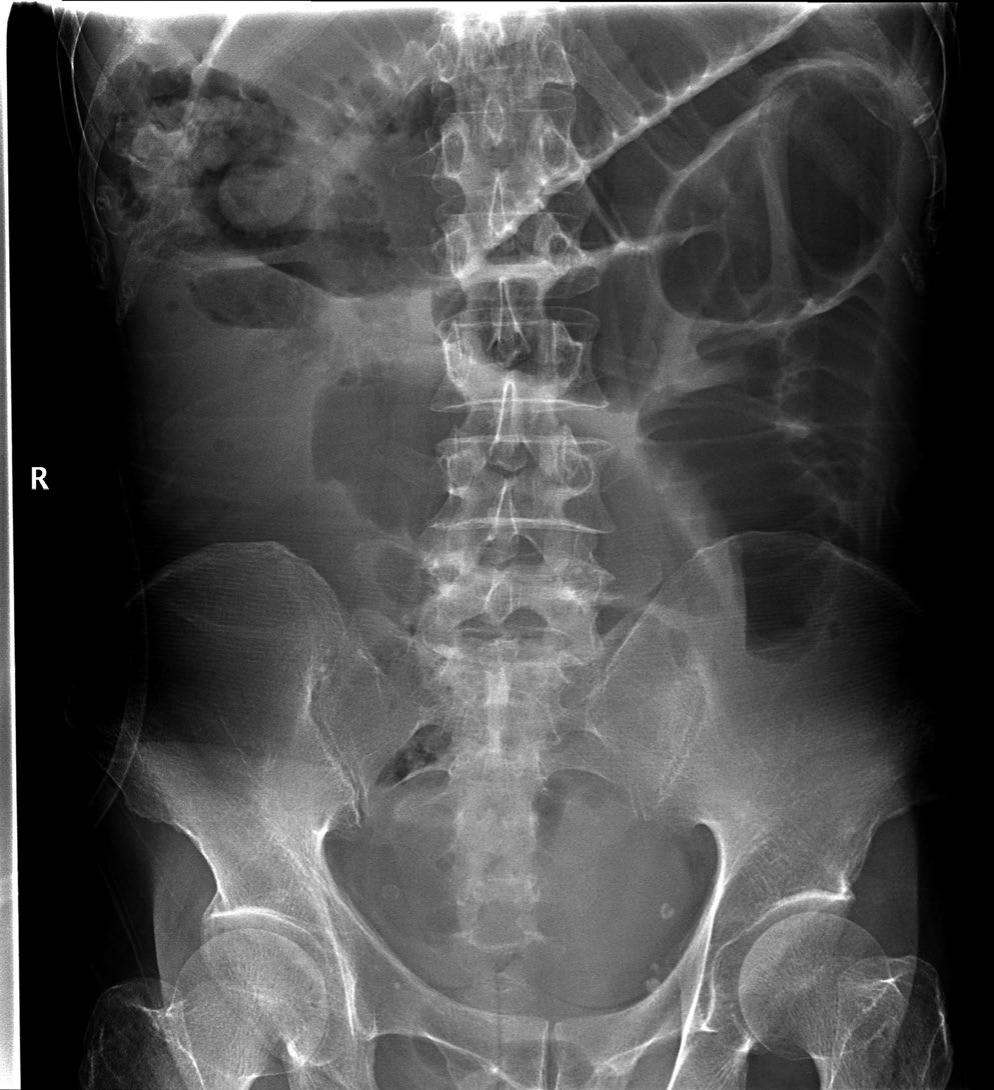
Abdominal X‐ray revealed an incomplete ileus

**Figure 4:**
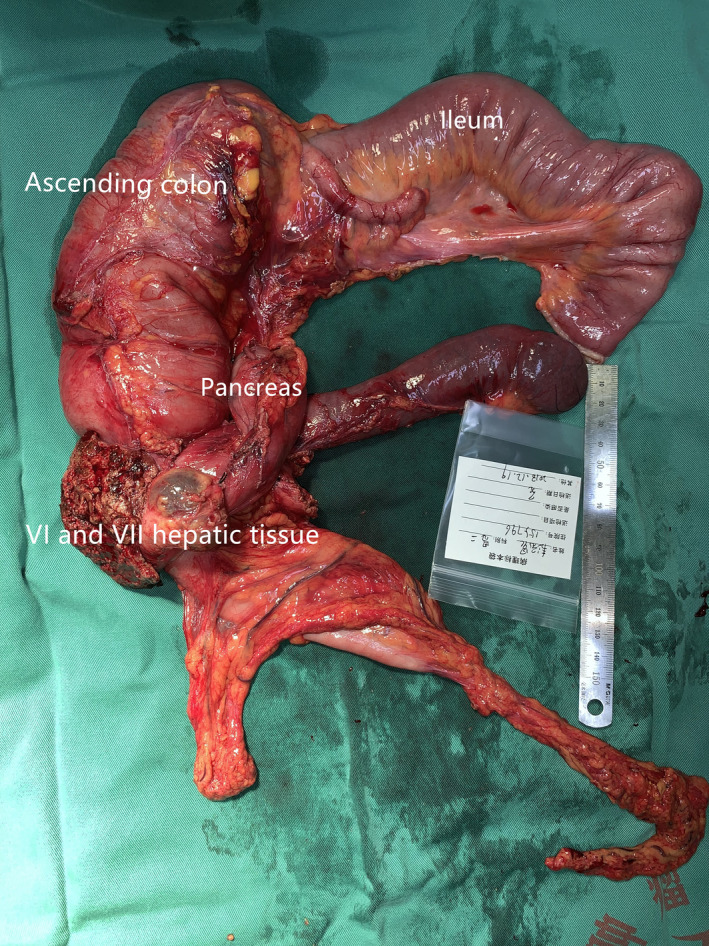
En bloc resection of the tumor

Our patient, a 54‐year‐old morbidly thin (body mass index 16.5) male, presented with a 2‐month history of hypodynamics, accompanied by poor appetite and weight loss. The patient's performance status was ECOG grade 1. Routine laboratory studies revealed microcytic anemia and preoperative computed tomography (CT) of the abdomen and pelvis revealed a tumor in the hepatic flexure of the colon involving segments VI and VII of the liver, gall bladder, and duodenum (Figure 1). Colonoscopy also identified a tumor located in the hepatic flexure of the colon (Figure 2). Biopsies of this mass revealed a medium to poorly differentiated tubular adenocarcinoma. The preoperative carcinoembryonic antigen level was 7.77 ng/mL, and the CA19‐9 level was 357.3 U/ml. The patient subsequently underwent 4 cycles of 5FU/oxaliplatin (FOLFOX regiment), followed by 4 months of follow‐up CT and colonoscope examinations, and the tumor was evaluated as stable disease (SD) （Figure 3). However, an abdominal X‐ray revealed an incomplete ileus (Figure 4). No tenderness or rebound was found in the patient.

We planned to provide immediate surgical treatment for the patient. However, the patient nutritional status was poor (NRS‐2002 grade 4)^3^ accompanied by hypoproteinemia (ALB: 29.9 g/L); therefore, the patient was preoperatively supplemented with human serum albumin and parenteral nutrition. This improved the patient's nutritional status, and he eventually underwent surgery.

## 2 SURGICAL PROCEDURES

The patient was placed in the supine, split leg position. After the operation, the position was adjusted to lower the head and foot height and raise the right and lower left side (Table 1).

**Table 1:** Postoperative value of amylopsin in drainage fluid

Postoperative day	Left abdominal drainage tube（U/L）	Right abdominal drainage tub（U/L）	T‐tube （U/L）		Blood （U/L）
1	514	980	‐		‐
2	130	861	16		84
3	29	119	7		45
4	29	119	7		‐
5	26	112	1		‐

The tumor was located in the hepatic flexure of the colon with adhesion to the right side of the descending part of the duodenum; it had invaded the right lobe of the liver. First, we performed a D3 expanded right hemicolectomy. During the procedure, we dissected the right hemicolon Toldt's fascia beside the ileocolonic vessels and in front of the superior mesenteric artery, confirming that the tumor had not invaded the superior mesenteric vein. Next, a duodenopancreatectomy was performed with en bloc resection and adequate free margins. After that, we dissected the right side of the hepatoduodenal ligament and, using a thumb, opened the hepatogastric ligament beside the hepatoduodenal ligament from left to right to determine if the tumor had invaded the right side of the portal vein. Finally, a sectional VI and VII hepatic segmentectomy en bloc resection was performed (Figure 4).

Postoperative pathology: 1. A (right semicolon) medium‐poorly differentiated tubular adenocarcinoma of mass infiltration type, sized 6 × 5 × 4.5 cm, and involving carcinoma tissue had infiltrated the whole layer of the intestinal wall and had broken through the serosal layer. The carcinoma tissue had invaded the liver, gallbladder, pancreas, and duodenum. A microscopic examination revealed a partial necrosis and focal calcification, as well as a vascular carcinoma thrombus and nerve invasion. Chronic inflammation of the colonic mucosa was noted; samples of the stomach, ileum, and appendix showed chronic inflammation of the mucosa, but no cancer. The pancreatic resection margin was positive, while the liver, stomach, duodenum, ileum, and colon were all negative. Four of 4 lymph nodes (12 groups) and 13 of 22 lymph nodes (mesenteric lymph nodes) were examined for metastatic carcinoma with a maximum diameter of 1.3 cm; an additional 2 nodules of carcinoma were noted.

We evaluated a postoperative pancreatic leak according to the International Study Group for Pancreatic Fistula (ISGPF).^4^ Postoperative amylase levels were continuously monitored in the abdominal drainage tube and t‐tube drainage fluid for 5 days and were evaluated as ISGPF classification A. The patient was advanced to a liquid diet on postoperative day (POD) 5 and was discharged on POD 15 without complaint. According to the National Comprehensive Cancer Network (NCCN) guidelines, this patient should be received remaining 8 cycles of 5FU/oxaliplatin (FOLFOX regiment).

Quality of life (QoL) assessment: We followed up the patient at 1, 3, and 6 months after surgery. The ECOG scale was used to evaluate the patient's function, which was 2, 2, and 3. It was found that the patient's physical functional status and independent activities gradually decreased, and his QoL gradually decreased.

At 1, 3, and 6 months after surgery, we used the EQ‐5D‐5L™ questionnaire (©EuroQol Group, Rotterdam, the Netherlands) ^5^to measure the QoL of our patient in the following areas of investigation (mobility, self‐care activities, usual activities, pain/discomfort, and anxiety/depression) with each dimension scored on 5 levels (ie, no problem, slight problem, moderate problem, severe problem, and extreme problem). The patient's average QoL scores were 76, 80, and 82.

### 2.1 Follow‐up

The patient received 2 cycles of 5FU/oxaliplatin (FOLFOX). After 6 months, tumors recurred in the patient's liver and lung. The disease‐free survival period for patient was 6 months. The patient had no chance to accept surgery, refused to undergo chemotherapy, and died 1 year after the surgery. The total days before he stay in hospital was 57 days.

## 3 DISCUSSION

Grey Turner in 1929 published the first report of a duodenal resection for locally advanced colon cancer, whereas Van Prohaska et al^6^ performed the first associated duodenopancreatectomy for direct colon cancer invasion in 1953. Since then, direct invasion of the pancreatic head and duodenum by a colonic tumor remains a major surgical challenge in the treatment of colorectal cancer.^7^, ^8^, ^9^ To date, several studies have reported the en bloc resection for T4B colorectal cancer that has invaded adjacent organs, such as the urinary bladder,^10^ anal sphincter,^11^ and liver.^12^

En bloc resection is sometimes required to cure T4B stage colorectal cancer.^13^ Many investigators have reported that this resection for colorectal cancer has acceptable morbidity and mortality rates and a fair long‐term prognosis;^14^, ^15^ therefore, they have emphasized the benefits of this procedure. For example, a study by Cihan Alalar^16^ showed that en bloc pancreaticoduodenectomy for locally advanced right colon cancers may result in long‐term survival with acceptable morbidity and mortality rates. However, a highly extended operation like an en bloc resection is also recognized to increase morbidity and mortality.^17^ Some large tumors that have invaded neighboring organs, such as the pelvic organs and pancreas, are unresectable because of the dangers posed by high surgical stress.^18^

Our case report suggests that a surgical approach for total resection of the tumor via the right colon is safe and, for patients with poor preoperative nutritional status who undergo perioperative parenteral nutrition support, may be a welcomed solution. The combination of the analyzed factors associated with the MVR or other factors related to the MVR, such as the number of anastomoses and width of dissection, could be speculatively indicated to affect the occurrence of complications.

Reducing the mortality and morbidity rate is challenging. Some studies have showed that en bloc resections increased the rates of infectious complications and ileus, but not other noninfectious complications.^19^ Other studies have shown that tumor size and depth of invasion, both of which were associated with en bloc resection, are independent risk factors, although in combination they might influence morbidity.^20^ In our case report, we found no postoperative morbidity, anastomotic leakage, or postoperative infection.

Preoperative neoadjuvant chemotherapy has been performed on patients, resulting in tumor contracture scars and intestinal cavity stenosis. For T4B stage CRC, whether patients may benefit from adjuvant chemotherapy remains controversial. Some studies showed neoadjuvant chemoradiotherapy may downstage some of the primary tumor, enhance the R0 resection rate, and reduce the recurrence rate.^21^ Others, however, reported that adjuvant chemotherapy has failed to bring unequivocal benefits to these patients, which leaves surgical radical resection as the sole option with a proven positive impact on the survival of these patients.^22^, ^23^, ^24^, ^25^ Thus, management of selected patients with T4B stage CRC remains controversial and may benefit from adjuvant treatment as recommended by the American Society of Clinical Oncology.^26^ For our patient, 4 cycles of adjuvant chemotherapy were carried out; however, we found the effect to be SD and an intestinal obstruction followed. Thus, surgery was performed.

For T4B colorectal cancer, we emphasize that en bloc resection of the tumor and the adjacent infiltrated organs is advisable in suitable patients in order to avoid jeopardizing complete tumor excision in a patient who has a realistic chance of being cured.^27^ The overall survival rates for T4B en bloc pancreaticoduodenectomy and right colectomy colorectal cancer patients are 72% at 1 year and 60% at 2 years.^28^ Recurrence was found in our patient in the liver and lung. He refused further treatment and died 1 year after surgery.

In conclusion, we describe our surgical approach for total resection of the tumor via the right colon to achieve the recommended oncologic margins for an en bloc resection of a locally advanced colon cancer discovered at exploration. To achieve better oncologic outcomes, en bloc colorectal cancer resection should be performed. Admittedly, some patients may be over‐treated by this aggressive approach; however, when practiced by experienced surgeons, en bloc resection usually carries no added morbidity and guarantees a better oncologic outcome.

## 4 CONCLUSIONS

En bloc resection for T4B patients has an acceptable survival rate of morbidity and mortality, but more case studies are needed concerning long‐term healing. In order to achieve a better oncologic outcome, multidisciplinary teamwork and multi‐modal treatment regimens can be utilized, and this procedure should be undertaken by a Noticed an experienced surgeon.

## CONFLICT OF INTEREST

None declared.

## AUTHOR CONTRIBUTIONS

All authors: involved in the preparation of this manuscript. LM and ZH: wrote the manuscript. LM: collected the tissue sample. HL: performed the histopathological investigations and revised the manuscript. JL, HZ, YL, and XM: performed clinical supervision of the case and revised the manuscript. All authors: read and approved the final manuscript.

## ETHICS APPROVAL AND CONSENT TO PARTICIPATE

Ethical approval was obtained from the ethics committee of Guangxi Cancer Hospital to report a case to describe the en bloc resection of a T4B stage cancer of the hepatic flexure of the colon invading the liver, gall bladder, and pancreas/duodenum (register number LW2019047). Informed consent was obtained from the patient for the case description and photograph material.

## CONSENT FOR PUBLICATION

We obtained the patient’s consent for publication.

## Data Availability

The datasets used and/or analyzed during the current study are available from the corresponding author on reasonable request.
